# How do stakeholders from multiple hierarchical levels of a large provincial health system define engagement? A qualitative study

**DOI:** 10.1186/s13012-017-0625-5

**Published:** 2017-08-01

**Authors:** Jill M. Norris, Deborah E. White, Lorelli Nowell, Kelly Mrklas, Henry T. Stelfox

**Affiliations:** 1 Faculty of Nursing, University of Calgary, 2500 University Drive NW, Calgary, AB T2N 1N4 Canada; 2 Research Priorities, Planning and Implementation, Alberta Health Services, 11th Floor, South Tower, Foothills Medical Centre, 1403–29 St. NW, Calgary, AB T2N 2T9 Canada; 3 Department of Community Health Sciences, Cumming School of Medicine, University of Calgary, Calgary, AB Canada; 4 O’Brien Institute of Public Health, Cumming School of Medicine, University of Calgary, Calgary, AB Canada

**Keywords:** Engagement, Clinical networks, Implementation, Quality improvement, Innovations, Health services, Organizational change, Involvement, Participation

## Abstract

**Background:**

Engaging stakeholders from varied organizational levels is essential to successful healthcare quality improvement. However, engagement has been hard to achieve and to measure across diverse stakeholders. Further, current implementation science models provide little clarity about what engagement means, despite its importance.

The aim of this study was to understand how stakeholders of healthcare improvement initiatives defined engagement.

**Methods:**

Participants (*n* = 86) in this qualitative thematic study were purposively sampled for individual interviews. Participants included leaders, core members, frontline clinicians, support personnel, and other stakeholders of Strategic Clinical Networks in Alberta Health Services, a Canadian provincial health system with over 108,000 employees. We used an iterative thematic approach to analyze participants’ responses to the question, “How do you define engagement?”

**Results:**

Regardless of their organizational role, participants defined engagement through three interrelated themes. First, engagement was active participation from willing and committed stakeholders, with levels that ranged from information sharing to full decision-making. Second, engagement centered on a shared decision-making process about meaningful change for everyone “around the table,” those who are most impacted. Third, engagement was two-way interactions that began early in the change process, where exchanges were respectful and all stakeholders felt heard and understood.

**Conclusions:**

This study highlights the commonalities of how stakeholders in a large healthcare system defined engagement—a shared understanding and terminology—to guide and improve stakeholder engagement. Overall, engagement was an active and committed decision-making about a meaningful problem through respectful interactions and dialog where everyone’s voice is considered. Our results may be used in conjunction with current implementation models to provide clarity about what engagement means and how to engage various stakeholders.

## Background

Stakeholder engagement has been described as essential and even “critical” [1–3] for moving knowledge into action within healthcare [4, 5]. Efforts to transform large systems are more successful when healthcare professionals are engaged, resulting in improvements to clinical outcomes and patient safety [6–9], quality of care [8, 10, 11], and financial performance [8]. While there is a growing body of best practices for patient [12–14] and physician engagement [7, 15–17], organizations remain challenged in engaging the wide range of professional groups who contribute to improving healthcare [18–20]. Engagement seldom occurs in isolation of one profession, but rather occurs across hierarchical organizational groups that vary in their power and status, worldviews, settings, and motives [21].

Though conceptualized in varied ways, engagement features prominently across the five categories of theoretical approaches to implementation science described by Nilsen [22]: classic theories, process models, determinant frameworks, implementation theories, and evaluation frameworks. Classic theories—those originating from disciplines outside of implementation science and applied to understand what influences implementation outcomes [22]—include theories of *employee* or *work engagement*. Employee engagement refers to a combination of psychological states (e.g., passion, commitment, willingness) and dispositions/traits (e.g., personality, conscientiousness) with observable behaviors (e.g., involvement, participation, action, focused efforts) [23–26]. More specifically, observable behaviors have been classified as adaptive and focused on desired organizational outcomes [24], including extra-role performance (proactivity, knowledge sharing, creativity, adaptability) [27], organizational citizenship behavior (discretionary effort) [26], and pro-change behaviors (initiating and supporting change initiatives) [28]. Employee engagement has been measured in efforts to understand how a large-scale improvement program influenced the vigor, dedication, and absorption [29] of hospital-based ward teams [30].

*Public participation* models [31] also have utility for conceptualizing engagement, given the evidence that healthcare stakeholders should be involved right from planning implementation through to sustaining and disseminating project findings [32–35]. Early models such as the Arnstein’s ladder of participation [36] (and later modifications [37, 38]) and the International Association of Public Participation (IAP2) spectrum of public participation [39] propose that organizational outcomes improve when stakeholders are included in setting priories and making decisions with increasing levels of engagement, or typologies of participation. Specifically, levels of engagement span from one-way information sharing to shared decision-making and stakeholder empowerment [40–42], a spectrum that involves increasingly participatory, transformative, and democratic processes [31]. While used to inform engagement with patient groups [43] and healthcare clinicians [44, 45], there is minimal empirical evidence to support the use of these models in implementation [46].

Other theoretical approaches [22] depict engagement as a process, a key ingredient or mechanism, as well as an outcome of implementation efforts without clearly defining the term *engagement* (see Table 1 for examples). These descriptions vary across and even within individual models. For example, the Consolidated Framework for Implementation Research (CFIR) [33] places engagement within several domains: within readiness for implementation in the inner setting (implementation context), the process of engaging (the actions of change), and could be also interpreted indirectly from the characteristics of individuals (e.g., individual identification with organization, knowledge and beliefs about the intervention) and other aspects of the outer setting domain (e.g., patient needs and resources, cosmopolitanism).

**Table 1:** Descriptions of engagement within select implementation models, theories, or frameworks

Description	Model or framework	Concept	Definition
Process	Stages of implementation completion [35]	Stage 1: *Engagement* (activities)	“Date site is informed services/program available…Date of interest indicated” (p. 3)
	CIHR model of knowledge translation [32]	Integrated knowledge translation	“*Involving* knowledge users as equal partners alongside researchers…Each stage in the research process is an opportunity for significant *collaboration* with knowledge users”
		Knowledge user	“A knowledge user’s level of *engagement* in the research process may vary in intensity and complexity depending on the nature of the research and on his/her information needs”
		Dissemination	“*Engaging* knowledge users in developing and executing dissemination/implementation plan”
	Quality implementation framework [34]	Phase 1: initial considerations (capacity-building strategies)	“Obtaining explicit *buy-in* from critical stakeholders and fostering a supportive community/organizational climate” (p. 468)
	CFIR [33]	Process	“Planning, *engaging*, executing, and reflecting and evaluating. These activities may be accomplished formally or informally through, for example, grassroots change efforts.” (p. 15)
		Process: *engaging*	“Attracting and involving appropriate individuals… through a combined strategy” (p. 11)
Mechanisms	CFIR [33]	Characteristics of individuals	Knowledge and beliefs, individual identification with organization, other *personal attributes*
		Inner setting: readiness for implementation	“Leadership *engagement*: commitment, involvement, and accountability of leaders and managers” (p. 9)
		Inner setting: implementation climate, compatibility	“The degree of tangible fit between *meaning and values* attached to the intervention by involved individuals, how those align with individuals’ own norms, values, and perceived risks and needs” (p. 8)
		Inner setting: implementation climate, learning climate	“A climate in which: leaders express their own fallibility and need for team members’ assistance and *input*; team members feel that they are essential, valued, and knowledgeable *partners* in the change process” (p. 9)
	COM-B [77]	Capability	“Capability is defined as the individual’s psychological and physical capacity to *engage* in the activity concerned.” (p. 4)
	Normalization process theory [78]	Cognitive participation	“Within the purposive interaction chains that make up an implementation process, a practice is framed through cognitive participation, the symbolic and real enrolments and *engagements* of human actors that position them for the interactional and material work of collective action.” (p. 543)
		Collective action	“This work may be to reshape behaviours or actions, to employ objects or artefacts, or it may be to reorganize relationships and contexts – but it involves collective *purposive action* aimed at some goal” (p. 544)
	Re-AIM [79]	Reach	“An individual-level measure (e.g., patient or employee) of *participation*. Reach refers to the percentage and risk characteristics of persons who receive or are affected by a policy or program.” (p. 1323)
Output	i-PARIHS [80]	Outcome: successful implementation	“Individuals, teams and stakeholders are *engaged*, motivated and ‘own’ the innovation” (p. 4)
	QUERI [81]	Dissemination	“An active, versus passive, effort to communicate tailored information to target audiences with the goal of *engagement* and information use”
	Organizational Readiness for Change [82]	Change-related efforts	“Members are more likely to initiate change (e.g., institute new policies, procedures, or practices), exert greater *effort* in support of change, and exhibit greater *persistence* in the face of obstacles or setbacks during implementation…. will exhibit more *pro-social, change-related behavior*” (p. 5)

Italics added for emphasis by authors

Given these diverse interpretations, we wondered how healthcare leaders and those diverse organizational groups with whom they aim to engage in dissemination and implementation—from frontline clinicians to business professionals, to patients and research analysts—defined (i.e., operationalized) engagement. To date, their perspectives have not been empirically investigated, which is an important first step towards creating a useful measure of engagement for tracking and subsequently improving engagement. Therefore, our study leveraged the opportunity to explore how healthcare stakeholders defined engagement within a new organization-wide, structural improvement initiative focused on the translation of evidence to practice in Alberta, Canada.

## Methods

### Setting

In 2012, Alberta Health Services established Strategic Clinical Networks (SCNs) in Alberta, Canada, to improve a broad range of healthcare delivery outcomes. Alberta Health Services is a provincial health system with over 108,000 employees providing healthcare services to a population of 4.1 million residents. We took advantage of this opportunity to study engagement in the newly formed SCNs as their mandate included (1) engaging partners throughout the health eco-system; (2) testing, spreading, and scaling of evidence-based practice; and (3) aligning with operational processes and geographical zones. Globally, these networks intend to facilitate engagement of stakeholders across multidisciplinary and hierarchical organizational levels. Each SCN is province-wide and has a core committee (approximately 35 individuals) designed to connect multiple stakeholder groups. Depending on the SCN, the core committee and project working groups include patients, clinicians, representatives from the five geographical care delivery zones and clinical operations, organizational experts (e.g., data acquisition, knowledge translation), leaders, researchers, and policy makers. At the time of the study, an adapted IAP2 spectrum [39] was used across Alberta Health Services to guide patient and clinician engagement strategies. In consultation with the study knowledge users, 9 of the 13 SCNs were purposively selected for participation in this study as they exhibited a range of maturity (i.e., the length of time established) and had projects that were implemented at the time of the study.

### Design

Our team used a qualitative thematic design [47] that was underpinned by pragmatic philosophy [48] and an integrated knowledge translation approach [49, 50]. Thematic analysis is a highly flexible methodology that can result in rich, complex accounts from different research participants, underlining similarities and differences, as well as generating unanticipated insights [47, 51]. Our team had pre-existing relationships with our knowledge users, and we collaborated throughout the research process. However, the leaders were not involved in data collection, data analysis, or drafting or approving the resulting manuscript. Data from individual interviews was collected between January and August 2014. Here, we present data from answers to a single introductory interview question “How do you define engagement?” and from the subsequent prompts to further clarify and expand upon the participants’ answers.

### Sampling and recruitment

Members of the SCNs and key organizational and operational leaders linked to the SCNs were eligible to participate in the study. Network members (SCN leads, core committee, working groups) were identified and recruited through membership lists. We also sampled key organizational leaders, given their central role in facilitating the implementation efforts of the SCNs. Network members were asked to identify these key leaders throughout the organization, who were subsequently approached by DW to participate in the study using snowball sampling. Each individual was emailed an invitation to participate in an interview. Purposive sampling for maximum variation was used to ensure that interviewees were recruited from each of the 9 SCNs, multiple geographic regions, professional roles, and roles within the SCNs. Sampling continued until data saturation was reached—the point at which no new themes emerged [52]. As a team, we assessed saturation using our auditable, structured codebook that noted changes of the coding framework [53].

### Data collection

After receiving written, informed consent, experienced interviewers (DW, KM, LN) completed semi-structured one-on-one interviews with stakeholders via telephone. Additional demographic information, such as age, professional experience, and role in the networks, were collected. The broader interview guide was pilot tested and was informed by our document review, the perspectives of knowledge users, and by the conceptual framework guiding our program of research. Interviews lasted 30–60 minutes and were digitally recorded, transcribed verbatim, and de-identified prior to analysis.

### Data analysis

All transcripts were assigned a unique identifier and imported into NVivo 10 for data management and analysis. Data was analyzed using an inductive thematic approach [47] to generate common, interactive themes involving coding, categorizing, and conceptualizing [52]. Coders first independently reviewed a sample of the transcripts and began to formulate provisional codes and themes. Bi-weekly coding meetings with the research team and experienced research assistants created a mutual understanding of codes and calibration and helped refine the coding framework. Teams of two coders examined and assigned sections of text to codes, representing themes or subthemes. Extracts of data were coded to as many themes/subthemes as relevant, and we wrote memos to record emerging impressions or interesting aspects of the data. Themes were further refined and reduced by examining coherent patterns in the coded data extracts. Using constant comparison [54], we conducted comparisons within the same group (hierarchical levels of the organization) and between different groups. A reference document defining each node of the coding framework was developed and modified to reflect coding discussions. Documentation and underlying rationale for changes to the framework were maintained to establish an audit trail.

### Rigor

We ensured rigor in conducting this study using Lincoln and Guba’s [55] criteria for trustworthiness: credibility, transferability, dependability, and confirmability [56] (Nowell LS, Norris JM, White DE, Moules NJ. “Thematic analysis: Striving to meet the trustworthiness criteria.” Int J Qual Methods. Submitted). Our team included researchers from nursing and medicine, all with a background in knowledge translation and health systems research. Three investigators were clinicians (physician, nurse). Our team meetings provided a venue for reflexivity and debriefing among the team, including intentionally exploring discrepant data and asking questions of our interpretations and stance. We maintained a detailed audit trail of all decisions, including a codebook, meeting minutes, and file naming conventions. The teams of the two researchers coded all transcripts, and decisions about themes and subthemes were vetted within the team. All names of themes and subthemes reflect the participants’ voice, and we returned to the raw data to further verify our results. Finally, we conducted member checking (respondent validation) through multiple presentations of our results to each SCN in the study and the AHS executive team.

## Results

### Sample characteristics

From 424 members of 9 SCNs, 138 members expressed interest in participating in an interview (33% response rate); 75 members were then purposefully selected for an interview, along with other organizational leaders (*n* = 11). Overall, 86 individuals from diverse backgrounds and settings were interviewed. Table 2 details stakeholder characteristics. Stakeholders from each of the 9 SCNs were represented and held multiple roles in the SCNs including leader/manager (27%), core or working group member (47%), patient representative (5%), and support personnel (13%). An additional 9% of stakeholders were geographic zone leaders for the broader organization. Over half of the sample were female (70%) and 40 to 59 years of age (57%), and 42% had 25+ years of professional experience. Stakeholders exhibited a variety of professional designations, including nurses (26%), physicians (14%), and executives (14%).

**Table 2:** Participant characteristics

Characteristics	*n*	%
Gender		
Female	60	70
Male	26	30
Age		
18–29 years	1	1
30–39 years	11	13
40–49 years	20	23
50–59 years	29	34
60+ years	10	12
Professional experience		
<5 years	7	8
10–14 years	9	11
15–19 years	9	11
20–24 years	9	11
25+ years	36	42
Professional designation		
Nurse	23	26
Physician	12	14
Executive	12	14
Other non-clinician	14	16
Researcher or analyst	7	8
Other allied health professionals	6	7
Occupational therapist	4	5
Manager, health services administration	3	4
Role within the SCN		
Leader, project manager	23	27
SCN member	40	47
SCN member—patient	4	5
Support personnel	11	13
Geographic zone leader	8	9
Focus of the SCN		
Cardiovascular and stroke	15	17
Surgery	13	15
Bone and joint	12	14
Diabetes, obesity, and nutrition	10	12
Seniors health	8	9
Critical care	5	6
Addictions and mental health	4	5
Cancer	4	5
Emergency	4	5
Leaders who worked across SCNs	11	13

### Initial commentary about the question

When asked to define engagement, stakeholders often prefaced their answer with a remark, including expressions of amusement and laughter.

Okay. [laughter] How long have you got? I can talk about engagement for a really long time. (P41, executive director)

Engagement was described as “a catch phrase” (P86, registered nurse) discussed and used extensively across the organization to the point of overuse. One participant described that the senior leadership “at every meeting recently, said they want to stop using the word because it’s kind of lost meaning. [laughter] They said because we use it for everything now and it’s kind of become diluted.” (P15, administrator).

Engagement was described as difficult or hard to define, with “no one answer to that” (P57, leader). Definitions differed “depending upon the context” (P41, executive director), “for different people, but it also means different things, depending on what you’re working on” (P86, registered nurse). Several stakeholders also emphasized the importance of having a clear definition to work from:

There’s not really a short and sweet answer. I think it needs to be co-defined and supported in its definition by the absolute top levels of government and our organization. So that you can just get on with it, because there’s been a lot of time spent on, you know, what is it? (P89, provincial quality improvement)

### Components of engagement

Participants provided examples of idealized engagement and how they had seen engagement previously enacted (both positive and negative). While we examined for differences between participants at different organizational levels, none were apparent. Themes elicited from stakeholder responses were categorized into three interrelated and broad themes (Table 3): (1) individual participation, (2) connecting around a purpose, and (3) meaningful interactions and dialog.

1. *Individual participation*

*Commitment and effort.* Many stakeholders defined engagement as a “commitment,” “investment,” “drive,” and “a passion.” These concepts were often paired with action or efforts: towards a goal, to working with other groups, and to moving things forward. One stakeholder conveyed that engagement is passion “and then actually doing something with it” (P13, nurse practitioner). Commitment was a defining characteristic of engagement but was also identified as a motivating factor for continued engagement and efforts. Engagement was seen to depend on professional responsibility or accountability for the work of the SCNs, beyond personal commitments.

*Individual willingness to participate.* Engagement required a personal choice or individual willingness to interact. Stakeholders emphasized that those who are seeking their involvement need to be willing to “consider,” “be open,” “listen,” “hear,” or “change.” Several stakeholders differentiated engagement between “those who really want to be here, versus those who are here for alternative reasons or maybe are not as invested in the mission or the organization” (P95, leader). Others emphasized that engagement meant that one was not being “volun-told”: “you’re the chosen one and you were told to participate” (P32, senior executive).

*Active participation.* Stakeholders further defined engagement as an active process (e.g., interaction, conversation, role). From one side, engagement was seen as actively soliciting and involving stakeholders; from the other, actively giving feedback and interacting “so that you’re not just a passive recipient of information.” (P1, medical director). Engagement also led to actions that resulted from “interactive engagement” (P8, patient researcher), including “parceling off some of the work and having individual contribution around that work to make the whole thing come together” (P47, patient advisor).

*Varying levels of involvement.* Involvement spanned many stakeholders’ definitions of engagement, including the “level” or “degree” to which stakeholders were involved. Involvement included participation in planning, projects, activities, and processes. Several stakeholders described levels of engagement, emphasizing the fact that “different levels are appropriate for different situations. Lower level engagement isn’t wrong, it’s just different” (P42, registered nurse). This “leveling” was often linked to the concept of managing the desires of stakeholders to be “involved at the level that they feel that they need to be involved” (P86, registered nurse) and also managing the expectations of stakeholders by clarifying “precisely what level of engagement you’re asking for as you go into any particular exercise” (P56, leader).

The IAP2 spectrum of engagement [39] was directly named and indirectly described throughout the interviews. One participant described it as “informing and then moving up through consulting, involving, collaborating and empowering” (P42, registered nurse) leading to higher levels of engagement. Stakeholders referred to it as “that engagement continuum,” “framework,” “IP2 scale,” or “engagement levels.” Several stakeholders also differentiated involvement from awareness or being informed, the knowledge of “what’s going on” that is removed from the intensity or effort that involvement required. When stakeholders detailed elements of the spectrum, lower levels of engagement started with informing as the first level of the spectrum, but there were variations of the last level—“accountability,” “ownership,” “empower,” “full decision-making involvement,” and “collaborating.”

2. *Connecting around a purpose*

*The focus for change is an interesting and relevant problem for us.* Engagement meant ensuring that the focus of change is relevant and meaningful and that those who are most impacted by it “see some value” in the issue. It also involved the action of “getting people interested” by linking the implementation efforts to their needs:

When I talk with frontline teams and frontline clinicians and staff it’s all about we are satisfying a fundamental need that you have and by doing so, you are providing back to us a tangible effort and participation in a real way (P18, project manager).

Stakeholders also acknowledged that individuals may not be interested, despite the importance of their perspective to implementation: “What’s their carrot?...The challenge in that, though, is their driver may not be your deliverable” (P80, project manager). For example, stakeholders repeatedly mentioned the multiple priorities and time pressures that frontline clinicians experienced and that if “they don’t see the value [of the initiative], then they’re not going to feel the support or enthusiastic or feel that the added effort and time is going to be worth it” (P43, administrator). Others spoke of stakeholders becoming disengaged or cynical, even destructive, without addressing a relevant problem:

If [stakeholders] just sign out because they can’t answer “what’s in it for me?”, then they disengage, and if you’re lucky that’s the best they do because disengage you can manage. Destructive behavior, derailing, and that kind of behavior is much harder to manage. (P43, administrator)

*Shared vision and decision-making.* Stakeholders viewed engagement as aligning or developing mutually defined goals. With the shared purpose, everyone needs “clarity as to what they are going to do and how it will be availed” (P33, physician), which included their scope, focus, intent, and ways to best work together to accomplish a shared mandate. Joint decision-making and negotiation also played into engagement. Several stakeholders mentioned that in some cases, involvement in decision-making occurred before projects were approved, including early adopter sites.

*A voice around the table.* Stakeholders frequently used the literal (i.e., meetings) and metaphorical (i.e., representation) description of being “around the table” within the context of engagement. To engage successfully, several stakeholders emphasized the need for the right representation for stakeholder groups and to determine “what everybody around the table can actually bring to achieve goals” (P52, physician), including their focus, intent, and knowledge. For stakeholder groups who were represented by an individual at meetings, several stakeholders expressed the importance that these representatives felt like they had “a voice at the table” (P41, executive director).

3. *Meaningful interaction and dialog*

*Two-way contribution, not a one-way information push.* Engagement was defined for many as a “bi-directional” or “two-way” dialog or process “where you’re both giving and receiving” (P14, business manager). Several stakeholders contrasted this to what engagement was not to them: “a one-way information push” (P91, provincial planning) or situations when information does not come back down the chain to stakeholders after they provided input, the “one-way piece” of engagement (P10, occupational therapist). Engagement should be “something more than just sending an e-mail and asking for information” (P36, director). Information or being informed was described as a lower form of engagement.

*Communication.* Stakeholders defined engagement as entering into iterative “discussions,” “dialog,” or “conversations.”

To me, engagement has a lot to do with communication. How are you communicating it? Where are you communicating it to? Are you getting it to multiple levels? (P90, patient care manager)

While there were “a lot of people having a lot of conversation in [the] networks,” the communication often felt top-down, driven by the leadership team rather than being initiated by the broader network community. Others described communication as an exercise in information sharing rather than “full decision-making involvement” (P56, senior executive).

The networks have, from what I’ve seen in the past, have been really focused on just communication and they don’t sort of go to that extent to figure out who their constituency actually is…they’ve been very passive. (P84, data analyst)

One participant (P36, director) provided an example of the need for conversations, not emails, to fully understand how they could apply best evidence to a clinical issue: “let’s say you’re in seniors care…you have to focus down to knowing are we talking about dementia patients, Alzheimer patients…long-term care facilities…acute care…You need to have a conversation to just nail down…what the problem statement is.”

*An invitation early in the process.* The act of “inviting,” “asking,” or “soliciting” was referred to by many stakeholders, and was often described in the context of requests for an interaction (dialog, feedback) or a contribution (guidance, support, or involvement). Ideally, an invitation would come “very early in the process” or “right from the get-go” to understand the issues and problems faced by stakeholders. One stakeholder (P2, data analyst) illustrated his ideal view of engagement from a past experience with a researcher who initiated a meaningful partnership with the unit by asking, “What would be things we would like to know more about? What would our priority questions be?” as compared to “researchers that are interested and here are the initiatives that they decided.” By asking what stakeholders needed early on (i.e., pull), partners were better able to attain “a full understanding of what could be done” (P47, patient advisor) instead of pushing an initiative that was not a good fit for the setting.

*Respect and sincerity*. With the diversity of stakeholder groups that SCNs need to work with, “symbiotic” and “respectful” interactions characterized many descriptions of engagement. Engagement also needed to embody the qualities of being “authentic,” “sincere,” or “genuine”—to be “transparent about what one is doing” (P76, medical director). Sincerity needed to be both communicated to and felt by stakeholders. One participant characterized engagement as being “genuinely…supported to be involved and heard. You feel that you’re being sincerely [heard]—your perspectives are being heard and being incorporated into the work” (P77, strategy lead). Others expressed that establishing trust was important to engagement: “you can establish rapport initially, but engagement is when you sort of cross the threshold into a trust-building relationship” (P5, coordinator).

*Listening and understanding; being heard and considered.* Stakeholders noted the importance of listening to input from multiple groups and that “the input is valued” (P112, data analyst) and ensuring that stakeholders “know their needs or their requirements are being considered and addressed” (P92, project manager). A number of stakeholders referred to ensuring that “voices are heard,” particularly during the early stages of project development. When voices were not heard, others described being “disengaged”: “if you’re going to put time in and resources and you’re going to rally the troops, and there’s no output, then you know I just sort of folded up my tent” (P9, medical director).

**Table 3:** Themes and subthemes

Theme	Subtheme	Exemplar quotes
Individual participation	Commitment and effort	“It’s having a personal commitment to what’s going on in your work world […] and having a vested interest and energy into doing something that contributes to that in a positive way, right? Putting effort forward to do better at whatever role you’ve got and how that best supports the system” (P115, patient care manager)
	Willingness to participate	“A willingness and motivation to participate” (P38, patient care manager)
	Active participation	“I really do see it as an active action. You know it can’t be passive and when you have sort of a passive frame of mind, then it becomes a committee meeting base and nothing gets done kind of a structure. So, that’s why I think the active piece is really important” (P74, research lead)
	Varying levels of involvement	“That model of engagement, you know, between inform and those five layers of things…when I think of engagement, I do actually think of that model” (P78, network manager)
Connecting around a purpose	An interesting and relevant problem	“It’s very important that the focus of this rally to be engaged is an important problem that a critical mass of people is going to come to. So it has to be a good idea, and someone has to pick a relevant, important idea, because nobody is going to come running to yet another discussion unless they feel that there’s a strong will and interest.” (P23, researcher)
	Shared vision and decision-making	“We have to develop a shared objective between the networks and the outside world, the health system and the users of the health system to what we want to do and where we want to go. So, that’s engagement. It’s formulating that shared vision.” (P93, manager)
	A voice around the table	“Nobody around the table is more important than another… We’re all on the same ground and … all of our opinions were important.” (P12, executive director)
Meaningful interaction and dialog	Two-way contribution, not a one-way information push	“It’s a two-way street, where I’m prepared, interested, knowledgeable, have different ideas or opinions that I want to either bring forward or share. And that on the flip side, they’re willing to hear what I have to say, willing to consider it, maybe alter some ideas” (P59, nurse practitioner)
	Communication	“Engagement is being able to truthfully communicate with stakeholders who will affected one way or the other by the particular project or study, by the behavior or actions that would come out of it. But to be able to have sincere communication back and forth and being able to listen in a respectful way” (P35, quality improvement)
	An invitation early in the process	“It comes back to number one, even being invited…when they looked at who the committee members needed to be…that was the beginning…right from the get-go they were engaging” (P30, clinical lead)
	Listening and understanding; being heard and considered	“Engagement to me is as much listening or, maybe at this stage in our evolution, to spend more time listening and understanding what others are doing in the area, what the issues are, where the opportunities might lay for SCNs to provide more guidance or leadership.” (P54, senior executive)
	Respect and sincerity	“The respectful inclusion of diverse perspectives to increase our effectiveness in terms of whether it’s decision-making, planning, evaluation.” (P117, patient experience)

## Discussion

**Fig. 1:**
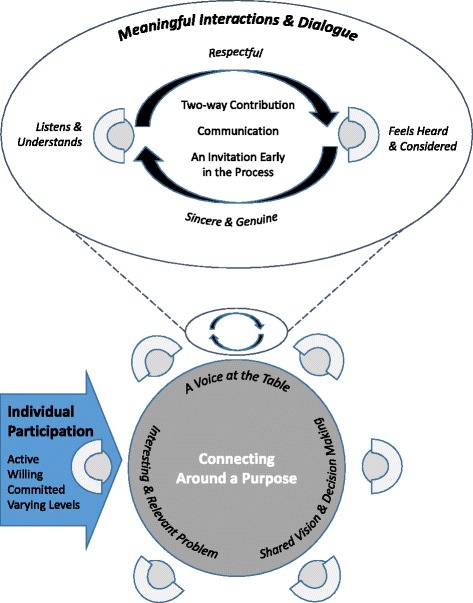
Components of engagement

Stakeholders in this study acknowledged extensive use of the term *engagement* across the health system and highlighted the need for a clear definition to guide further conversations and actions towards improving engagement. Regardless of the stakeholders’ organizational role, engagement was expressed as three interrelated components. First, engagement was described as active participation from willing and committed stakeholders, the levels of which ranged from information sharing to more extensive involvement such as collaboration or full decision-making. Second, engagement involved shared focus and decision-making around relevant change to the stakeholders. Third, interactions between stakeholders were described as requiring two-way communication, initiated early in the change process, and that were respectful and approached with sincerity, where all stakeholders felt, heard, and understood. Figure 1 illustrates our conceptualization of these themes. Based on our data, we propose the following definition of engagement: active and committed decision-making about a meaningful problem through respectful interactions and dialog where everyone’s voice is considered.

Within our program of research, SCN members at different organizational levels significantly differed in their views of the *levels* at which they were engaged: leaders were more engaged, while frontline clinicians were less engaged [46]. In this study, however, stakeholders across these same organizational groups *defined engagement* similarly—an unexpected finding. This suggests that there may be a common vocabulary and framework from which to initiate engagement across healthcare organizations and systems.

The theme *individual participation* reflects the definition of medical engagement put forth by Spurgeon et al. ([17], p. 214): “the active and positive contribution of doctors within their normal working roles to maintaining and enhancing the performance of the organisation.” It is also consistent with the CFIR [33] domain *characteristics of individuals* and the multidimensional employee engagement construct from organizational research, which represents the notion of an individual *being* engaged in change [23–26]. In contrast, stakeholders in our study predominantly referred to the *action* of engaging others (with the goal for individuals to *be* engaged) [23]. This is an important distinction because it implies that engagement is a process or series of actions (arguably, an intervention) and an antecedent to engagement (as a state of being or mechanism). Engagement is not static but rather a process that requires cultivation over time. Like the predominant discourse throughout the implementation science literature, engagement was seen by stakeholders as inviting people to come together to participate across phases of healthcare improvement, from early priority setting to sustainment of initiatives.

An important outcome of this study is the descriptions of what engagement is not: engagement is not one-way communication. This finding, however, is not completely resolved within our data as some participants considered sharing information as engagement within the context of the IAP2 spectrum of engagement [39]. Employees who have adequate information about organizational changes are more aware of what is coming and how change will impact the organization, resulting in less uncertainty and stress, more openness for change, and a higher perceived need for change [57]. Moreover, information about organizational change impacts how trustworthy healthcare professionals perceive senior management to be [58]. Indeed, awareness/knowledge is a domain within CFIR and the Theoretical Domains Framework [59]. We argue, though, that despite the importance of communicating change information (dissemination), it is a separate process from engagement. Participants often passionately detailed how one-way information pushes—emails, talking *to* (rather than *with*) people, *telling* people how, when, or who—were not engagement. Further, by failing to create a reciprocal avenue for feedback or greater involvement, network members and stakeholders may have disengaged or even demonstrated outward expressions of dissatisfaction such as being destructive or derailing an initiative. In several models of participation, information sharing is labeled as tokenism or passive participation: stakeholders participate in change by being told or educated by those in power, without an ability to have a voice nor assurances that their views will be considered [31]. In a study of 750 healthcare professionals, involvement in organizational change reduced cynicism towards change; conversely, sharing information did not consistently influence cynicism [58].

Diverging from predominant conceptualizations of engagement, the *meaningful interaction and dialog* theme closely replicate the construct of *respectful engagement* described by Dutton [60] within the positive organizational literature. Respectful engagement is defined as interpersonal interactions or behaviors that convey respect, positive regard, and worth within an organization or team [61, 62]. Respectful engagement involves five actions: (1) conveying presence through body language and availability; (2) being genuine; (3) communicating affirmation by recognizing someone’s situation and value, by expressing interest and by considering someone’s time; (4) effective listening; and (5) supportive communication, which involves requests and not demands and communicating in specific and descriptive ways [62]. This concept is distinct from the concepts of mutual respect (a domain of relational quality) and organizational respect, which can be understood as high status within the organization [63] or as perceptions of how the collective and individuals are treated with respect in an organization [64, 65]. Recent research suggests that respectful engagement reaches beyond courteous conversations. Positive emotions that come with respectful engagement such as feeling valued and accepted can motivate organizational members to have productive ongoing interactions with their colleagues about their goals and work (termed *relational information processing*), which further promotes creativity [60].

Together, we note that these findings are congruent with necessary aspects of meaningful partnership described for researcher-knowledge user relationships in the knowledge translation and implementation literature. Similarities include a deliberate focus on active and participatory engagement, on issues of mutual concern to knowledge users and their contexts, and on collaborative decision-making processes aimed at knowledge co-production and use [5, 66–69]. While integrated knowledge translation is an approach in which research is developed and executed within the implementation context [32, 70, 71], it fosters a more fully integrated model for implementation advancing questions and improvement initiatives of mutual interest; knowledge users are active participants throughout the process (i.e., priority setting, designing the initiative and implementation). This approach to implementation also draws our attention to the internal organizational context as well as external context and their influence on implementation efforts and engagement of individuals within these contexts. There may be something to be gained from this angle on engagement, within healthcare structures or systems that are deliberately focused on increasing the uptake and use of evidence into policy and practice to improve patient care, healthcare services, and the sustainability of the healthcare system [72].

### Implications

The three components of engagement identified in this study could apply to other settings that require interprofessional collaboration, perspectives of multiple stakeholders, or effective teamwork across hierarchical organizational levels. Quality improvement initiatives in health care, education, and business are examples. Our study suggests that co-constructed interactions between stakeholders be made explicit in future improvement models, particularly those focused on knowledge translation as part of their mandate. This will require moving beyond traditional communication strategies to engagement processes that intentionally target the psychological characteristics and adaptable behaviors we desire from stakeholders [24, 26, 73]. One potential avenue towards this could include considering the role of social exchange theory, particularly leader-member exchange theory to further explore the relational components of engagement. With others [31, 74–76], we call for further refining the concept of engagement (both as a state and a process) including the timing, purpose, locus, organizational context, and the actors within the process (as well as those who are excluded) as these domains are largely unexplored territories.

### Strengths and limitations

The transferability of study findings is strengthened by the inclusion of participants from a diverse range of healthcare professions and roles within a large healthcare organization (i.e., maximum variation), including patients who were involved in the networks. This allowed us to explore the complexity and variation of engagement to achieve the fullest understanding of this concept, fostering a broader applicability. However, the results need to be interpreted within the context of the study’s limitations, including reporting the perceptions of stakeholders about how engagement was defined and not how it occurs within the organization. We were limited to sampling stakeholders of 9 of the 13 SCNs in a provincial healthcare system. To enhance the transferability of our study findings, we included quotes and interpretations that aimed to sufficiently illuminate the organizational context. Comparison with stakeholders in other healthcare organizations or disciplines would strengthen the research, as would exploring the perspectives from a larger sample of patients. In this study, we did not see distinctions in the definition of engagement by role in the network, including the views of patient representatives.

## Conclusions

Engagement of stakeholders is essential for successful healthcare quality improvement yet is difficult to achieve and measure. Current implementation science models identify engagement as important while providing little clarity about what engagement means or how to engage various stakeholders in implementing innovations. This study provides an in-depth description of how multiple stakeholders of a large healthcare organization focused on improving the uptake and use of evidence-defined engagement. Our results may be used in conjunction with improvement models to understand, develop, and guide engagement strategies.

## Abbreviations

AHS: Alberta Health Services
CFIR: Consolidated Framework for Implementation Research
IAP2: International Association for Public Participation
SCN: Strategic Clinical Networks
